# MiR-130a is aberrantly overexpressed in adult acute myeloid leukemia with t(8;21) and its suppression induces AML cell death

**DOI:** 10.1080/03009734.2018.1440037

**Published:** 2018-03-01

**Authors:** Chao Ding, Su-Ning Chen, Roderick A. F. Macleod, Hans G. Drexler, Stefan Nagel, De-Pei Wu, Ai-Ning Sun, Hai-Ping Dai

**Affiliations:** a Jiangsu Institute of Hematology, The First Affiliated Hospital of Soochow University, Suzhou, People’s Republic of China; b Collaborative Innovation Center of Hematology, Soochow University, Suzhou, People’s Republic of China; c Leibniz Institute DSMZ–German Collection of Microorganisms and Cell Cultures, Braunschweig, Germany; d Suzhou Institute of Blood and Marrow Transplantation, Soochow University, Suzhou, People’s Republic of China

**Keywords:** Acute myeloid leukemia, miR-130a, t(8;21*)*, outcome, overexpression

## Abstract

**Background:**

Emerging evidence has revealed that miRNAs can function as oncogenes or tumor suppressor genes in leukemia. The ectopic expression of miR-130a has been reported in chronic leukemia, but our understanding of the biological implications of miR-130a expression remains incomplete.

**Methods:**

We quantified a cohort of *de novo* acute myeloid leukemia (AML) by bead-based miRNA and real-time quantitative PCR (Rq-PCR). The luciferase reporter gene assay was analyzed after the plasmid constructs which contain 5’-UTR of miR-130a and a Renilla luciferase reporter plasmid were transfected simultaneously into 293T cells. MTT and caspase 3/7 apoptosis assays were used to test cell viability and apoptosis.

**Results:**

We identified miR-130a as significantly overexpressed in t(8;21) AML. Expression of miR-130a decreased significantly once patients with t(8;21) achieved complete remission, but increased sharply at the time of relapse. In patients with t(8;21) AML, KIT mutational status was associated with miR-130a expression—with higher expression associated with KIT activating mutations. Increased miR-130a expression in t(8;21) AML was associated with slightly worse event-free survival; however, no impact on overall survival was observed. Knockdown of AML1/ETO protein in the SKNO-1 cell line resulted in decrease of expression of miR-130a. Direct binding of AML1/ETO fusion protein with the promoter sequence of miR-130a was detected with luciferase reporter gene assay. Following miR-130a knockdown, SKNO-1 demonstrated increased sensitivity to etoposide.

**Conclusions:**

Our data suggest that miR-130a is directly activated by AML1/ETO, and may act as a factor which is associated with leukemia burden, event-free survival, and chemotherapy sensitivity in t(8;21) AML.

## Introduction

Acute myeloid leukemia (AML) with t(8;21) accounts for 10%–20% of *de novo* AML patients, most of whom have favorable prognosis. However, a small proportion of AML patients with t(8;21) have relatively worse outcome due to secondary molecular genetic aberrations, including somatic mutations of *KIT* and *FLT3* (1,2). In recent years, it has been suggested that miRNA expression may be regulated by fusion proteins resulting from chromosome translocations such as t(8;21) (3,4).

MiRNAs are endogenous 19–25-nucleotide long non-coding RNAs which play important regulatory roles in cell proliferation, differentiation, and apoptosis (5). Emerging evidence has revealed that miRNAs represent a potential new class of tumor suppressors or oncogenes (6,7). As previously described, miR-126 is known to be overexpressed in t(8;21) AML, and can enhance proliferation and colony-forming/replating capacity of mouse normal bone marrow progenitor cells in concert with AML1/ETO (8). Overexpression of miR-126-5p was shown to be associated with drug resistance to cytarabine and poor prognosis in AML (9). In contrast, miR-193a was shown to be silenced via chromatin changes induced by AML1/ETO, enhancing the oncogenic activity of this fusion by repressing the expression of multiple target genes, such as *DNMT3A*, *HDAC3*, *KIT*, *CCND1*, and *MDM2* (10). While the importance of miRNAs in AML with AML1/ETO fusion gene has been suggested, the biological and clinical significance of microRNA deregulation in this subgroup remains poorly understood.

Chinese AML patients have relatively higher occurrence of t(8;21) as compared with the incidence in Western countries, 22.1% versus 8.8% in AML-M2 patients (11,12). As such, the primary aim of this study was to explore aberrantly expressed miRNAs in t(8;21) AML patients from China and to assess their potential contributions to leukemogenesis. To achieve our objectives, we analyzed the miRNA expression profiles in 156 *de novo* AML patients using a miRNA array. We validated our findings through the expression of miR-130a in primary bone marrow (BM) samples of patients with AML.

## Materials and methods

### Clinical samples

All primary samples were obtained from the Jiangsu Institute of Hematology (JIH) from 26 September 2005 to 25 September 2010, and collected after informed consent according to the Declaration of Helsinki and agreement by the Ethics Committee of the First Affiliated Hospital of Soochow University. For the miRNA profiling, BM samples from 156 *de novo* AML patients were collected at diagnosis. The clinical data were obtained from 10 May 2012 to 26 January 2014, and we had access to identifying information during this period under the permission of the Ethics Committee of the First Affiliated Hospital of Soochow University. The longest follow-up was 100 months. Among this cohort, 20 specimens were diagnosed as having core binding factor (CBF) AML, 16 with t(8;21) AML and 4 with inv(16) AML (Table 1). To evaluate the expression of miR-130a, BM samples from a non-overlapping cohort of 79 AML patients were collected. Among these, 32 had t(8;21) (including 10 paired samples taken at diagnosis and the first complete remission; 2 paired samples taken at diagnosis, complete remission, and the first morphological relapse; and 3 paired samples taken at diagnosis, partial remission, and complete remission), 11 had inv(16), 6 had t(15;17), 13 had rearrangements involving MLL, 10 had other aberrations, and 7 had normal karyotype (Table 2). In addition, BM samples from 10 healthy volunteers were chosen as negative controls. AML diagnosis was made according to the French-American-British (FAB) classification and revised with the World Health Organization (WHO 2008) classification criteria.

**Table 1.** Cytogenetic characteristics of 156 AML bone marrow samples for the miRNA array.

Cytogenetic group	Number of cases (%)	FAB subgroup (% of successful cases)							
		M0	M1	M2	M3	M4	M5	M6	Others
Successful	156	1	30	39	36	21	23	5	1
Abnormal	86 (55.1)	1 (100)	8 (26.7)	20 (51.3)	34 (94.4)	11 (52.4)	11 (47.8)	1 (20)	0 (0)
Favorable	52 (33.3)	0 (0)	2 (6.7)	12 (30.8)	32 (88.9)	6 (28.6)	0 (0)	0 (0)	0 (0)
t(15;17)	32 (20.5)	0 (0)	0 (0)	0 (0)	32 (88.9)	0 (0)	0 (0)	0 (0)	0 (0)
t(8;21)	16 (10.3)	0 (0)	2 (6.7)	12 (30.8)	0 (0)	2 (9.5)	0 (0)	0 (0)	0 (0)
inv(16)	4 (2.6)	0 (0)	0 (0)	0 (0)	0 (0)	4 (19)	0 (0)	0 (0)	0 (0)
Intermediate	94 (60.3)	1 (100)	27 (90)	25 (64.1)	3 (8.3)	14 (66.7)	19 (82.6)	4 (80)	1 (100)
Adverse	10 (6.4)	0 (0)	1 (3.3)	2 (5.1)	1 (2.8)	1 (4.8)	4 (17.4)	1 (20)	0 (0)

**Table 2.** Clinical characteristics of 79 AML patients for the detection of miR-130a.

Cytogenetic group	Number of cases (%)	Gender (M/F)	Median age (year)	Median WBC (×10^9^/L)	Median blasts (%)
t(8;21)	32	21/11	29.5	16.6	58
inv(16)	11	5/6	31	68.8	51
t(15;17)	6	3/3	34	24.3	80
Rearrangements involving *MLL*	13	4/9	37	13.96	75
Other abnormalities	10	5/5	56	92.25	69.25
Normal karyotype	7	3/4	35	31.4	91.4

### Cell lines and transfections

Authenticated cell lines were cultured as described previously, and were obtained from the DSMZ (Braunschweig, Germany) between November 2013 and September 2014 (13). Catalog numbers of the cell lines are as follows: HeLa (ACC-57), HL-60 (ACC-3), KASUMI-1 (ACC-220), KG-1 (ACC-14), ME-1 (ACC-537), ML-2 (ACC-15), MOLM-20 (ACC-591), MONOMAC-6 (ACC-124), MV4-11 (ACC-102), SHI-1 (ACC-645), SKNO-1 (ACC-690), THP-1 (ACC-16), U-937 (ACC-5). The siRNA oligonucleotides targeting AML1/ETO fusion gene were designed as described and obtained from MWG Eurofins (Ebersberg, Germany) (14). The pRedAML1/ETO plasmid and the control vectors were described previously (15). The miR-130a inhibitor and the miScript inhibitor negative control were obtained from Qiagen (Hilden, Germany). The siRNAs and the miRNA inhibitor were transfected into different cell lines by electroporation using the EPI-2500 impulse generator (Fisher, Heidelberg, Germany) at 350 V for 10 ms. The pRedAML1/ETO plasmid and the control vector were transiently transfected into HeLa cells using lipofectamine 2000 (Thermo Fisher Scientific, Dreieich, Germany), respectively. After 48 h, transfection efficiency was evaluated by western blot.

### MicroRNA array

Ficoll-separated BM mononuclear cells were collected from the patients at diagnosis. Total RNA was extracted using Trizol reagent (Invitrogen, Karlsruhe, Germany). MicroRNA from cell lines was extracted with the miRNeasy mini kit (Qiagen). Total RNA and miRNA were quantified by Nanodrop-1000 (Thermo Scientific). Bead-based miRNA expression profiling was applied for quantification of patients’ miRNA expression, performed at the Department of Genetics and Yale Stem Cell Center from Yale University (16). For quantification of miRNA expression of cell lines via profiling, Affymetrix GeneChip miRNA 2.0 system (High Wycombe, UK) was used. Analysis of expression data was performed using Microsoft Excel and online programs. We generated heat maps using CLUSTER version 2.11 and TREEVIEW version 1.60 (http://rana.lbl.gov/EisenSoftware.html).

### Real-time quantitative PCR (Rq-PCR)

The cDNA was reverse transcribed using the Taqman^®^ MicroRNA Reverse Transcription Kit (Applied Biosystems, Darmstadt, Germany). Real-time quantitative Rq-PCR was performed by the 7500 real-time system, using commercial buffers and primer sets (Applied Biosystems). For normalization of expression levels, we used U6 snRNA.

### Protein analysis

Western blot analysis was performed using a semi-dry method. Nuclear proteins were extracted with NE-PER Nuclear and Cytoplasmic Extractions Reagents (Thermo Fisher). Proteins obtained from cell lysates were transferred onto nitrocellulose membranes (Bio-Rad, München, Germany), which were blocked with 5% bovine serum albumin (BSA) dissolved in tris-buffered-saline buffer (TBS). The following antibodies were obtained from Cell Signaling Technology (Beverly, MA, USA): AML1 (4334), GAPDH (5174).

### Cloning procedures and reporter gene analysis

The 5’-UTR of miR-130a (−2058∼−1 and −2022∼−1, respectively) which contains the *AML1* consensus sequence TGTGGT was synthesized and cloned into the GV354 vector (MCS-firefly_Luc-PloyA-Tk-Renilla_Luc-PolyA, Genechem, Shanghai, China), respectively. Plasmid constructs with specified mutations were also synthesized and cloned into GV354 as controls. The plasmid constructs and the negative control plasmid were transfected simultaneously into 293T cells together with the pRedAML1/ETO plasmid and a Renilla luciferase reporter plasmid using the X-tremeGene HP DNA transfection reagent (Roche, Indianapolis, IN, USA). Cells were harvested after 48 h. Then luciferase activity was detected using the Dual Luciferase Reporter Assay System (Promega, Madison, WI, USA). The transfection assay was performed three times.

### Cell viability and apoptosis assay

Cell line SKNO-1 was plated at a concentration of 10,000 cells per well in triplicate for each condition in a 96-well plate 24 h after electroporation with the miR-130a inhibitor or the control miRNA inhibitor (Qiagen). Etoposide (5 μM, Sigma, München, Germany) or the same volume of DMSO (Sigma) (mock treatment) was added to each well. After 24 h, MTT (5 μg/mL, Sigma) was added to each well. After a 3-h incubation in 37 °C, reactions were stopped by adding DMSO/isopropanol (2:1). Absorbance was determined at 570 nm by an ELISA reader (Thermo Electron, Vantaa, Finland). For apoptosis, caspase 3/7 activity was detected using the ApoONE Homogeneous Caspase 3/7 Assay (Promega) following the manufacturer’s instruction. Fluorescence was measured by a spectrofluorometer instrument (Perkin Elmer, Waltham, MA, USA). The cell viability and apoptosis assays were performed in triplicate.

### Data analysis

The relative amount of miR-130a was calculated using the term 2^−ΔCT^; ΔCT was calculated by subtracting the Ct for U6 small nuclear RNA from those for miR-130a. Correlations between miR-130a expression and disease subtype were assessed using Mann–Whitney non-parametric tests. Correlations between miRNA expression and *KIT* mutational status was assessed using chi-square test. Overall survival (OS) and event-free survival for bone marrow test abnormality (EFS) were estimated using the Kaplan–Meier method and were compared by log-rank tests. The paired *t* test was applied for determining the difference of the luciferase activity, as well as change of inhibition rate and activity of caspase 3/7. All statistical analyses were performed with the SPSS 17.0 software. Values of *P* < 0.05 were deemed to have attained statistical significance.

## Results

### MiRNA array data

**Figure 1.**
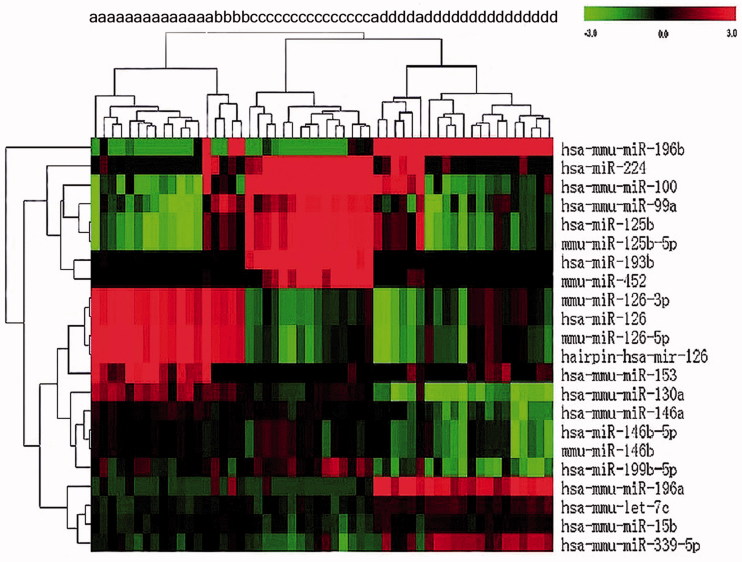
Hierarchical clustering of miRNA expression in a selected group of 54 AML patients utilizing 22 twofold differentially expressed miRNAs. MiRNA levels are shown as a heat map. (a) t(8;21); (b) inv(16): (c) t(15;17); (d) NK.

MiRNA expression data demonstrated that AML patients in different cytogenetic primary rearrangement classes had distinct miRNA expression profiles (Figure 1). Expression of miR-126-3p, miR-126-5p, miR-153, miR-130a, and miR-146a was significantly upregulated in AML with t(8;21) as compared with AML with t(15;17) or a normal karyotype. Conversely, let-7c, miR-15b, miR-99a, miR-100, miR-125b, miR-193b, miR-196a, miR-196b, miR-199b, miR-224, miR-339, and miR-452 were downregulated in t(8;21) compared with other cytogenetic groups (Figure 1).

### MiR-130a is overexpressed in cell lines and patients with the t(8;21) translocation

**Figure 2.**
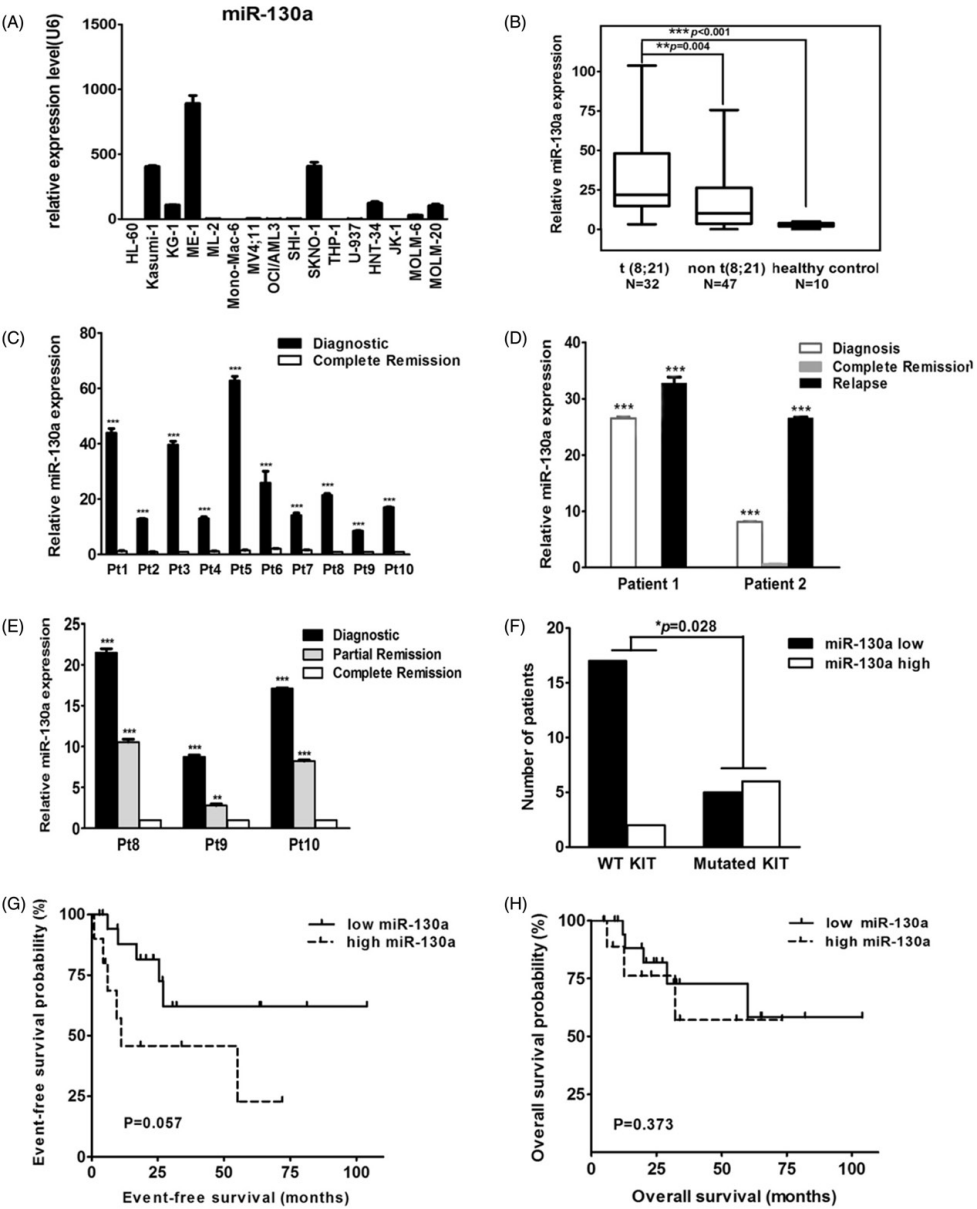
(A) Rq-PCR results of miR-130a expression in 16 AML cell lines, which reveal that the KASUMI-1 and SKNO-1 cell lines with t(8;21) and the ME-1 cell line with inv(16) had much higher expression of miR-130a when compared with other AML cell lines. (B) Rq-PCR results of miR-130a expression in 79 patients with AML and 10 healthy controls, which show that expression of miR-130a was significantly higher in primary BM samples with t(8;21) than in those with other cytogenetic characteristics and normal controls. (C) Rq-PCR results of miR-130a expression in 10 AML patients with t(8;21). The results demonstrate that all patients had much higher miR-130a expression level at diagnosis than at complete remission (CR). (D) Rq-PCR results of miR-130a expression in two AML patients with t(8;21). The results reveal that expression of miR-130a decreased significantly at the time of CR compared with that detected at diagnosis, contrasting with a sharp increase in the relapsed sample (***P < 0.01). (E) Rq-PCR results of miR-130a expression in three AML patients with t(8;21). The results show that expression of miR-130a decreased gradually both at the time of partial remission (PR) and CR compared with that detected at diagnosis. (F) Expression of miR-130a in AML patients according to mutational status of KIT. The results show that patients with KIT mutation had much higher overexpression of miR-130a (*P* = 0.028). (G) Event-free survival (EFS) of AML patients with t(8;21) in this study. The results reveal that t(8;21) patients with lower miR-130a expression had slightly better EFS compared with those had higher miR-130a expression (*P* = 0.057). (H) Overall survival (OS) of AML patients with t(8;21) in this study. The results reveal that expression level of miR-130a showed no effect on OS of t(8;21) AML (*P* = 0.373).

Expression of miR-130a was detected in 16 AML cell lines with different cytogenetic characteristics. Expression of let-7c and miR-196b was detected in nine AML cell lines. Only miR-130a was shown to be specifically upregulated in two cell lines harboring t(8;21): KASUMI-1 and SKNO-1 (Figure 2(A) and data not shown). Rq-PCR results revealed significantly elevated expression of miR-130a in patients with t(8;21) AML as compared with those that had other cytogenetic aberrations (*P* = 0.004) or healthy controls (*P* < 0.001) (Figure 2(B)). In 10 patients with t(8;21) AML, we observed a decrease of miR-130a expression at complete remission as compared with those levels at diagnosis (*P* < 0.001) (Figure 2(C)). In 2 of the 10 patients, expression of miR-130a increased sharply at relapse, to levels significantly higher than those detected at diagnosis (*P* < 0.001) (Figure 2(D)). In another three paired samples, we observed that the expression level of miR-130a decreased gradually in partial remission and in complete remission (Figure 2(E)). The median relative expression level of miR-130a in t(8;21) AML patients with wild-type *KIT* was 9.864 (1.575 ∼ 26.506) which is much lower than that detected in t(8;21) AML patients with mutated *KIT* (31.181, 7.875 ∼ 50.586). In a cohort of 32 t(8;21) AML patients with known *KIT* mutational status, 89.47% (17/19) of those with wild-type *KIT* were found to have lower expression of miR-130a as compared with the median value of overall miR-130a expression of the 32 t(8;21) patients. This ratio dropped to 45.45% (5/11) in t(8;21) AML patients with activating mutated *KIT* (7 with D816V, 2 with N822K, 1 with D816H, and 1 with D816Y) (Figure 2(F)). Survival analysis revealed that t(8;21) AML patients with high miR-130a expression demonstrated a slightly worse event-free survival trend compared with those with low expression of miR-130a (Figure 2(G)), while overall survival was unaffected (Figure 2(H)).

### MiR-130a was directly activated by the AML1/ETO fusion gene

**Figure 3.**
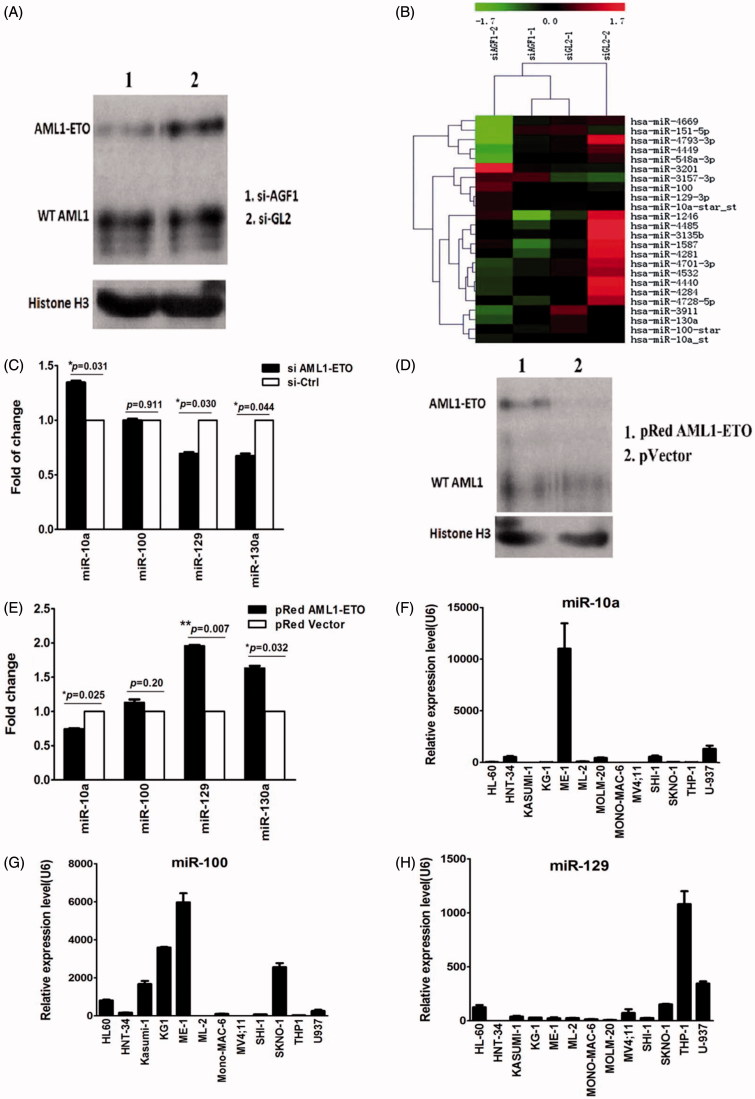
(A) Western blot results of expression of AML1/ETO in SKNO-1 cells. The results indicate that expression of the AML1/ETO fusion protein decreased significantly in SKNO-1 cells transfected with siRNA targeting AML1/ETO. SiAGF1 was a siRNA targeting the AML1/ETO fusion gene. SiGL2 was a siRNA targeting P pyralis luciferase, which was used as a negative control siRNA in this study. (B) Heat map of the miRNA array of SKNO-1 cells transfected with the AML1/ETO siRNA and the control siRNA. Levels of 24 differentially expressed miRNAs between the two groups are shown as a heat map. Two paired miRNA samples collected at two independent knockdown experiments were used. (C) Change of expression of four miRNAs in SKNO-1 with and without AML1/ETO knockdown. The results show increased expression of miR-10a, contrasting with decreased expression of miR-129 and miR-130a in SKNO-1 transfected with AML1/ETO siRNA when compared to controls. Standard errors are indicated. (D) Western blot results of expression of AML1/ETO in HeLa cells. The results indicate that AML1/ETO fusion protein was successfully overexpressed in HeLa cells transfected with the AML1/ETO plasmid compared with that transfected with the control vector. (E) Change of expression of four miRNAs in HeLa cells with and without AML1/ETO overexpression. The results show decreased expression of miR-10a, but increased expression of miR-129 and miR-130a in HeLa with AML1/ETO overexpression. Standard errors are indicated. (F, G, H) Expression of miR-10a, miR-100, and miR-129 in AML cell lines. The results demonstrate that these miRNAs are not uniquely upregulated or downregulated in AML cell lines with the t(8;21) translocation (KASUMI-1 and SKNO-1).

In SKNO-1 cells transiently transfected with the siRNA targeting the AML1/ETO, the efficacy of silencing was evaluated by western blot analysis of AML1/ETO protein as compared with mock-treated cells (Figure 3(A)). Global microRNA expression changes were evaluated using microRNA arrays in SKNO-1 cells with and without AML1/ETO knockdown. We detected a decrease of expression of miR-129 and miR-130a and an increase of miR-10a in SKNO-1 cells with AML1/ETO knockdown as compared with the controls, but miR-100 was not significantly affected (Figure 3(B)). These findings were confirmed with Rq-PCR, using SKNO-1 cells transfected with the above siRNAs (Figure 3(C)).

HeLa cells were transfected with either a plasmid containing full-length AML1/ETO cDNA or a control vector. Overexpression of AML1/ETO in HeLa was confirmed by western blot (Figure 3(D)). In HeLa cells with AML1/ETO overexpression, we detected upregulation of miR-129 and miR-130a, while a concomitant downregulation of miR-10a was observed by Rq-PCR; the change of expression of miR-100 was not statistically significant (Figure 3(E)). Furthermore, we examined expression of miR-10a, miR-100, and miR-129 in 13 AML cell lines with Rq-PCR. These results demonstrate that these three microRNAs are not uniquely upregulated or downregulated in SKNO-1 and KASUMI-1 cells which both had t(8;21) (Figure 3(F–H)). This suggests that they were not directly regulated by AML1/ETO as is miR-130a.

**Figure 4.**
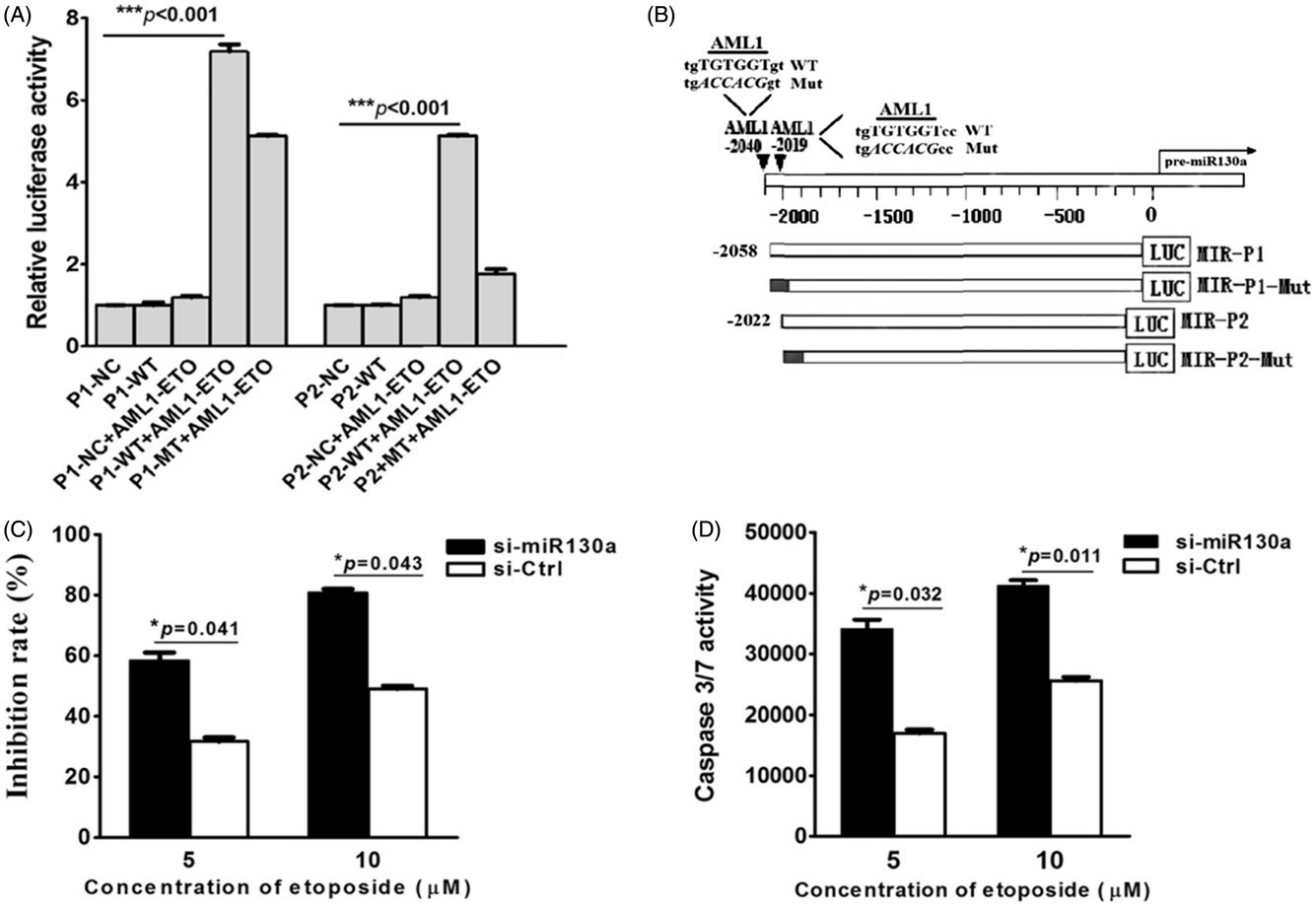
(A) Luciferase reporter gene assay. The results show that AML1/ETO could activate expression of vector containing the wild-type P1, the P1 mutant, and the wild-type P2, which could not activate the P2 mutant of the 5’-UTR sequence of pre-miR-130a. (NC = negative control luciferase reporter plasmid; P1-WT, P2-WT = luciferase plasmid containing the wild-type P1 or P2 sequence; P1-Mut, P2-Mut = luciferase plasmid containing the mutated P1 or P2 sequence; black = mutated regions). (B) Schematic structure of the luciferase plasmid construct. The results represent the average of three independent evaluations. (C) Growth inhibition by etoposide (5 μM) of transfected SKNO-1 cells after 24 h treatment. The results reveal that growth of SKNO-1 cells was obviously inhibited by etoposide compared to that transfected with the negative control miRNA inhibitor. (D) Caspase 3/7 activity in transfected SKNO-1 cells treated with different concentrations (5 μM and 10 μM) of etoposide for 24 h. The results show that the activity of caspase 3/7 increased significantly in SKNO-1 cells transfected with the miR-130a inhibitor compared to that transfected with the negative control inhibitor (*P = 0.032 and *P = 0.011, respectively).

Two *AML1* consensus sequences of TGTGGT (−2040∼−2035 and −2019∼−2014) were found in the 5’-UTR of miR-130a. Plasmids containing the wild-type (named P1-WT and P2-WT, respectively) and mutated sequences of these promoters (named P1-Mut and P2-Mut, respectively) were constructed. Results of the luciferase reporter gene analysis demonstrated that AML1/ETO could activate the wild-type P1 (*P* < 0.001), the mutated P1, and the wild-type P2 promoters, but could not activate the mutated P2 promoter (Figure 4(A,B)). These results suggest that either P1 or P2 is sufficient for regulation of miR-130a.

### MiR-130a sensitizes SKNO-1 cells to etoposide-induced cytotoxicity and apoptosis

Etoposide was used to treat SKNO-1 cells which had been transfected with miR-130a inhibitor or a control miRNA inhibitor. When compared with the control cells, SNKO-1 transfected with the miR-130a inhibitor showed a marked etoposide concentration-dependent increase in growth inhibition (Figure 4(C)). There was a statistically significant increase in caspase 3/7 activity in an etoposide concentration-dependent manner (Figure 4(D)).

## Discussion

In the present study, we describe the identification of miR-130a overexpression in *de novo* AML patients with t(8;21) and demonstrate the contributions of this miR-species on leukemogenesis. Across hematologic malignancies, our understanding of the biological implications of miR-130a expression remains incomplete. In chronic leukemia, downregulation of miR-130a has been reported, and as such the suggestion that miR-130a could be a tumor suppressor gene had been postulated (17,18). Importantly, the association between miR-130a expression and fusion proteins was first proposed by Suresh and collaborators who confirmed that miR-130a was downregulated in a BCR-ABL-dependent manner in chronic myelogenous leukemia (CML) when compared with normal controls (19). Reduced miR-130a expression resulted in repressed *CCND3* expression and evasion of negative growth regulation in leukemic cells. Similarly, in chronic lymphoid leukemia, miR-130a was also widely downregulated, while ectopic re-expression could modulate cell survival programs by regulating autophagic flux through downregulating *ATG2B* and *DICER* (18).

In comparison with these studies, high endogenous expression of miR-130a was initially detected in the t(8;21)(q22;q22) cell line Kasumi-1 by Häger et al. (20). Downregulation of miR-130a by antisense RNA rendered Kasumi-1 susceptible for TGF-β-induced cell growth inhibition (20). In accordance with the above findings, our results described the identification of miR-130a overexpression in t(8;21) AML, which was further confirmed to be activated by AML1/ETO. Based on our findings, we suggest that miR-130a changes disease characteristics in order to aggravate disease outcome in t(8;21) AML. In keeping with these findings, expression of miR-130a was significantly decreased in partial remission and once morphologically complete remission was achieved, while a sharp increase in miR-130a expression was observed at the time of relapse. Together these results suggest that miR-130a expression could be an indicator of leukemic burden in t(8;21) AML. However, we only examined two patients with miR-130a at diagnosis, remission, and relapse in this study. Further studies designed to specifically address these questions should be performed in an effort to characterize further the diagnostic and prognostic utility of miR-130a in t(8;21) AML.

Abnormal expression of miRNA may represent an important molecular marker to infer cancer prognosis. Thus, overexpression of miR-125b-2 in *ETV6/RUNX1* ALL provides a survival advantage to growth inhibitory signals in a p53-independent manner (21). More directly related to our findings, miR-130a was shown to be upregulated in gastric cancer, while reduced expression predicts longer overall survival in this disease (21,22). In the present study, we demonstrated that increased expression of miR-130a (above the average value) conferred slightly longer EFS in t(8;21), while it had no effect on OS. The t(8;21) AML patients with high miR-130 expression have an inclination to have *KIT* mutation (Figure 2(F)), and most of them received allogenic stem cell transplantation after standard induction and two cycles of consolidation chemotherapy consisting of high-dose cytarabine. That would partially explain why these patients had relatively lower EFS compared with those who only received chemotherapy. Because of the limited number of cases presented in this study, it is necessary to confirm these observations in more samples.

Normally, miRNAs inhibit protein translation or induce mRNA degradation through an imperfect or perfect base pair binding to the 3’-untranslated sequences (UTR) of target mRNAs (9). The imperfect matching between miRNAs and their targets implies that each miRNA can bind multiple mRNAs. Presently, *ATGB2*, *DICER1*, and *SMAD4* were all reported to be targets of miR-130a in different leukemic cells (18,20). According to the MIRTARBASE (http://mirtarbase.mbc.nctu.edu.tw), *HOXA5*, *HOXA10*, and *RUNX3* are predicted to be potential miR-130a targets, while targeting of *RUNX3* has been confirmed using luciferase reporter assay and miRNA array in gastric cancer (22). However, we were unable to detect any change of the above targets with western blot in SKNO-1 cells transfected with miR-130a inhibitor when compared with controls (data not shown). Thus, we believe that *HOXA5*, *HOXA10*, and *RUNX3* are unlikely to be targets of miR-130a in SKNO-1 cells.

MiRNAs may impact the prognosis of leukemia by modulating the drug sensitivity of leukemia cells. Shibayama and colleagues found that ectopically overexpressing miR-126-5p in KG-1 cells decreased sensitivity to cytarabine (9). Similarly, forced expression of miR-126 was demonstrated to inhibit apoptosis and increase the viability of AML cells (7,8). Following the methods described by Chen and colleagues, we treated SKNO-1 cells with etoposide following transfection with a miR-130a inhibitor (8). Our results reveal that SKNO-1 cells transfected with miR-130a inhibitor were more sensitive to etoposide showing higher induction of caspase 3/7 than controls. Thus, we postulate that overexpression of miR-130a could protect SKNO-1 cells from the cytotoxicity of etoposide through inhibiting activation of caspase 3/7. We hope that the above results may be further confirmed *in vivo* using animal models in the near future.

In this study, we demonstrated that miR-130a is aberrantly overexpressed in t(8;21) AML, and may be a direct transcriptional target of the AML1/ETO fusion gene. Expression of miR-130a varied significantly in different stages of t(8;21) AML, which could be used as indicators for leukemia burden, minimal residual disease, as well as event-free survival. MiR-130a was also found to provide a survival advantage by caspase activation in response to growth inhibitory conditions. Thus, we suggest that miR-130a may act as an adverse outcome predictor and a therapeutic target in t(8;21) AML.

In conclusion, our data indicate that miR-130a may contribute to the pathogenesis of t(8;21) AML. Further studies may clarify the prognostic significance of miR-130a and evaluate possible therapeutic interventions on the basis of this molecular change.
